# Shedding Light on the Elusive Role of Endothelial Cells in Cytomegalovirus Dissemination

**DOI:** 10.1371/journal.ppat.1002366

**Published:** 2011-11-17

**Authors:** Torsten Sacher, Joachim Andrassy, Aivars Kalnins, Lars Dölken, Stefan Jordan, Jürgen Podlech, Zsolt Ruzsics, Karl-Walter Jauch, Matthias J. Reddehase, Ulrich H. Koszinowski

**Affiliations:** 1 Max von Pettenkofer-Institute, Ludwig Maximilians-University, Munich, Germany; 2 Surgery Department, Klinikum Großhadern, Ludwig Maximilians-University, Munich, Germany; 3 Institute for Virology, University Medical Center of the Johannes Gutenberg-University Mainz, Mainz, Germany; Oregon Health and Science University, United States of America

## Abstract

Cytomegalovirus (CMV) is frequently transmitted by solid organ transplantation and is associated with graft failure. By forming the boundary between circulation and organ parenchyma, endothelial cells (EC) are suited for bidirectional virus spread from and to the transplant. We applied Cre/loxP-mediated green-fluorescence-tagging of EC-derived murine CMV (MCMV) to quantify the role of infected EC in transplantation-associated CMV dissemination in the mouse model. Both EC- and non-EC-derived virus originating from infected Tie2-*cre* ^+^ heart and kidney transplants were readily transmitted to MCMV-naïve recipients by primary viremia. In contrast, when a Tie2-*cre* ^+^ transplant was infected by primary viremia in an infected recipient, the recombined EC-derived virus poorly spread to recipient tissues. Similarly, in reverse direction, EC-derived virus from infected Tie2-*cre* ^+^ recipient tissues poorly spread to the transplant. These data contradict any privileged role of EC in CMV dissemination and challenge an indiscriminate applicability of the primary and secondary viremia concept of virus dissemination.

## Author Summary

More than sixty years ago Frank Fenner proposed that virus dissemination during acute infection originates from organs replicating virus to high titer (often liver or spleen) early in infection. Although never formally proven, this model has become commonly accepted and was applied to acute virus infections in general. Recently, we challenged this model by showing that - during acute murine cytomegalovirus infection – hepatocyte-derived virus hardly disseminates to other organs. We now applied our well established model of Cre/loxP-mediated green-fluorescence-tagging of MCMV to determine and quantify the role of infected endothelial cells (EC) in transplantation-associated CMV dissemination. We observed an only very poor dissemination of MCMV from the transplant to recipient tissues and vice versa. Interestingly, we observed no evidence for preferential dissemination of EC-derived virus. Significant differences in virus organ titers were found when comparing intravenous infection with transplant-mediated infection. This suggests a preferential dissemination of cell-associated virus in the transplant setting. In summary, our findings argue for a preferential dissemination of cell-associated MCMV but demonstrate that the Fenner model does not apply to MCMV.

## Introduction

Human Cytomegalovirus (HCMV), a member of the betaherpesvirus subfamily, represents an important opportunistic viral pathogen in the immune compromised host. Fetuses, AIDS patients, and recipients of both bone marrow and solid organ transplants are at high risk for the development of debilitating and potentially life-threatening CMV disease. Depending on the risk constellation and immunosuppressive regimen, CMV disease can occur in up to 60% of heart or kidney transplant recipients. Therefore, HCMV is the most important viral pathogen especially during the first six months after transplantation [1], [2]. The large variety of symptoms results from the broad cell and organ tropism of the virus [3], [4]. In addition, the virus is able to disseminate via blood [5]. According to Fenner (1949) a virus enters - after initial replication at the entry site (epithelia or transplant) - the blood stream and disseminates throughout the body to distal organs via a so-called primary viremia, which was confirmed to apply also to HCMV and MCMV [6], [7]. It is proposed that progeny virus from such organs can re-enter the blood circulation leading to a secondary viremia [6], [7] thus increasing the risk for widespread dissemination.

Leukocyte depletion of blood products derived from seropositive donors prior to transfusion efficiently prevents transfer of CMV to seronegative recipients [8], [9] indicating that virus present in blood is predominantly cell associated. The cell types responsible for this dissemination are of particular interest. Three kinds of cells have been suggested to be involved in virus dissemination via blood. All of them have been shown to be able to transfer infectious virus *ex vivo*: polymorphonuclear leukocytes (PMNL), monocytes/macrophages, and detached infected vascular endothelial cells (EC). Although PMNL are thought to be only abortively infected, they might still function as vehicles for infectious virus [10]. Circulating infected monocytes become permissive upon differentiation into tissue macrophages and may then release infectious progeny within target organs [11]. For example, rat CMV was transferred via *in vitro* infected granulocytes or monocytes [12]. Vascular EC are suggested to play an important role in CMV dissemination, and unique genetic features govern the CMV - EC interaction [13]. EC support productive infection and may detach upon infection thus serving as shuttles for the virus to other organs via the blood stream [14], [15], [16]. EC are permissive for HCMV *in vitro* [3] and are commonly found to be infected in tissue samples from both immune compromised patients [17] and mice [18]. In addition, EC support latent infection with the potential to reactivate CMV [19] and to start a new episode of infection. Notably, HCMV infection is a risk factor for restenosis after coronary atherectomy [20] and accelerates atherosclerosis following cardiac transplantation [21]. The anatomical position of EC lining blood vessels implies a bidirectional role in virus entry into and exit from the blood circulation and therefore might define the ability of viruses in general to disseminate via blood. In fact, HCMV-infected EC can protrude from the wall into the lumen of the blood vessels in patients with active cytomegalovirus infection [16]. Furthermore, circulating giant endothelial cells were found in blood samples of transplant patients [14] suggesting detachment of infected EC from the vessel wall and dissemination of HCMV via EC throughout the body.

Despite the undisputed and unique potential of EC in CMV infection and pathogenesis, it is still unknown whether infected EC are responsible for systemic virus dissemination during primary infection, contribute to this process, or merely represent an epiphenomenon with no causal involvement in the pathogenesis of organ disease [22]. Quantitative aspects of the contribution of infected EC to virus dissemination in the transplant situation are scarce and the presence of infected EC in the circulating blood does not prove that infected EC or HCMV produced by EC contribute or even govern virus dissemination from one site or organ to another.

To quantify and to address the fate of virus produced by specific cells, we developed a Cre/loxP mediated approach to label virus in defined cell types *in vivo* and then trace the viral progeny of that cell type [23], [24]. Cre recombinase recognizes two adjacent loxP sites and deletes the intervening DNA sequence. This reaction can remove a transcriptional stop signal between promoter and coding sequence resulting in gene expression. To study the role of EC in MCMV replication an MCMV mutant was used that contains a Cre-inducible *egfp* expression cassette (MCMV-*flox*). Mice expressing Cre recombinase under control of either the Tie2 or the Tek promoter, which is selectively expressed in vascular EC (Tie2-*cre* and Tek-*cre* mice), were infected with MCMV-flox. In this *in vivo* infection model MCMV-flox is efficiently recombined resulting in MCMV-rec only during virus replication in EC. It is important to note that Cre-mediated recombination of MCMV-flox is equally efficient in Tie2- and Tek-*cre* mice and only mediated by EC - as shown using bone marrow chimeras - thus providing highly concordant results by both mouse strains [24]. The resulting recombination is then stably maintained in the viral genome of the virus progeny.

Vascular EC are present in all organs. A way to study the role of EC in virus dissemination from one organ to another is to either introduce organs from an EC *cre*-negative donor mouse into an EC *cre-*positive host or vice versa. Here, we investigated export of EC-derived virus from heart and kidney transplants to recipients as well as import of EC-derived virus from recipients into heart transplants. This was achieved by counting and comparing the contribution of EGFP-positive EC-derived progeny to the total virus load of organs and tissues.

EC-derived virus from infected heart or kidney transplants readily disseminated to organs of MCMV-naïve recipients. The bulk of virus produced in and disseminated from heart is EC-derived, whereas in kidneys infected EC only provide a minor contribution. Yet, we found no evidence for any preferential dissemination of EC-derived virus from both types of transplants to other organs. The heart transplant was also tested as a target organ of EC-derived virus produced in recipient tissues. To our surprise, in contrast to the strong dissemination of virus originating from an infected transplant there was only minimal seeding of host EC-derived virus progeny to the transplant. Interestingly, this was independent of whether transplantation was performed prior to or after systemic host infection. In summary, our data argue against a privileged role of EC in virus dissemination.

## Results

### Virus dissemination from infected hearts into non-infected recipients

**Figure 1:**
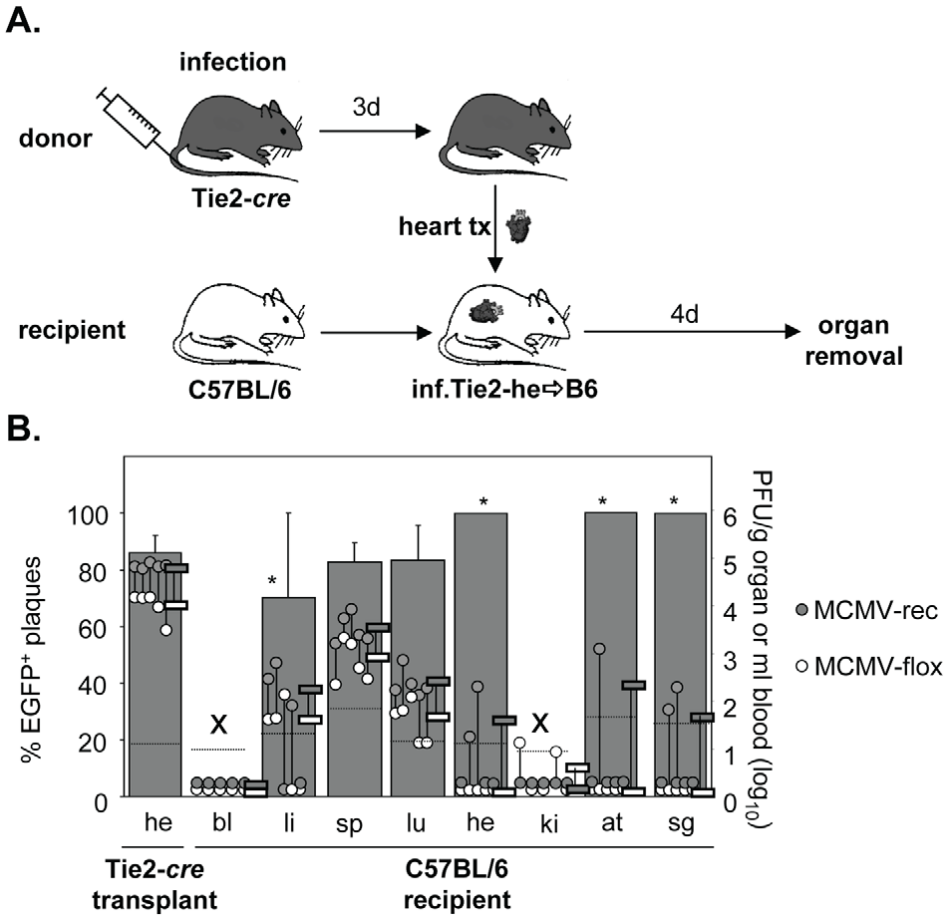
Dissemination of EC- and non-EC-derived MCMV from heart transplants to non-infected recipients via primary viremia. **A**. Tie2-*cre* mice (n = 5) were infected i.v. with 8×10^5^ PFU MCMV-flox. Three days after infection, hearts were transplanted into non-infected C57BL/6 mice. Four days after transplantation blood was taken and organs were collected from recipients to determine the contents of MCMV-flox and MCMV-rec plaque forming units (PFU) in various organs by plaque assay. **B**. The graph depicts virus load of MCMV-flox (open circles) and MCMV-rec (grey circles) per gram organ or ml blood for individual mice referring to the logarithmic scale on the right hand side. Open and grey circles are connected via a vertical line indicating that these data are derived from the same individual mouse. Horizontal bars mark mean values of absolute amounts of MCMV-rec (grey) and MCMV-flox (open). Dotted horizontal lines give detection limits of absolute amounts of PFU per gram organ or ml blood. Grey columns refer to the linear scale on the left hand side of the graph and show the mean percentage of EGFP^+^ plaques (MCMV-rec compared to MCMV-rec plus MCMV-flox), with the standard deviation indicated by vertical bars. Columns labeled with asterisks are calculated for virus-containing organs only. In both blood and kidney (x) virus titers where too low to reliably calculate the contribution of EC-derived virus. Abbreviations: he = heart; bl = blood; li = liver; sp = spleen; lu = lungs; ki = kidney; at = adipose tissue; sg = salivary glands.

Transplantation of organs from HCMV seropositive donors to seronegative recipients (D+/R-) is a known situation in transplantation medicine and represents the “high risk constellation” because up to 60% of the recipients can develop CMV disease [25]. In this D+R- setting CMV disease is caused by dissemination of HCMV from the transplanted organ to the recipient causing systemic symptoms with multiple organs being involved. The cellular source of disseminated virus has not been addressed, yet virus dissemination from infected heart transplants has also been described in the mouse model [26], [27]. To investigate whether and to which extent virus derived from EC of the transplant disseminates to organs of uninfected recipients, hearts from acutely infected Tie2-*cre* mice were transplanted heterotopically into non-infected syngeneic C57BL/6 mice (Fig. 1A). Four days after transplantation mice were sacrificed and organs collected to determine the amounts of non-recombined (non-EC-derived) and recombined (EC-derived) virus. In the heart transplant, high virus loads (∼10^5^ PFU/g organ) of predominantly recombined virus (∼85%) were observed, confirming that the transplantation procedure itself did not affect MCMV replication in general and demonstrating a very high recombination efficiency (Fig. 1B). This is in accordance with high recombination efficiency observed previously for heart and lungs of Tie2-*cre* mice [24]. Virus titers in different organs of mice infected via the heart transplant were 10 to 10,000-fold lower than generally seen following systemic (i.v.) infection with ∼1×10^6^ PFU [24]. The relative amounts of MCMV-rec and MCMV-flox in the recipient organs, however, essentially reflected the situation in the heart transplant, with some minor variance. Thus, EC-derived virus virtually disseminated equally well as non-EC-derived virus from the heart transplant.

### Virus dissemination from infected kidneys into non-infected recipients

**Figure 2:**
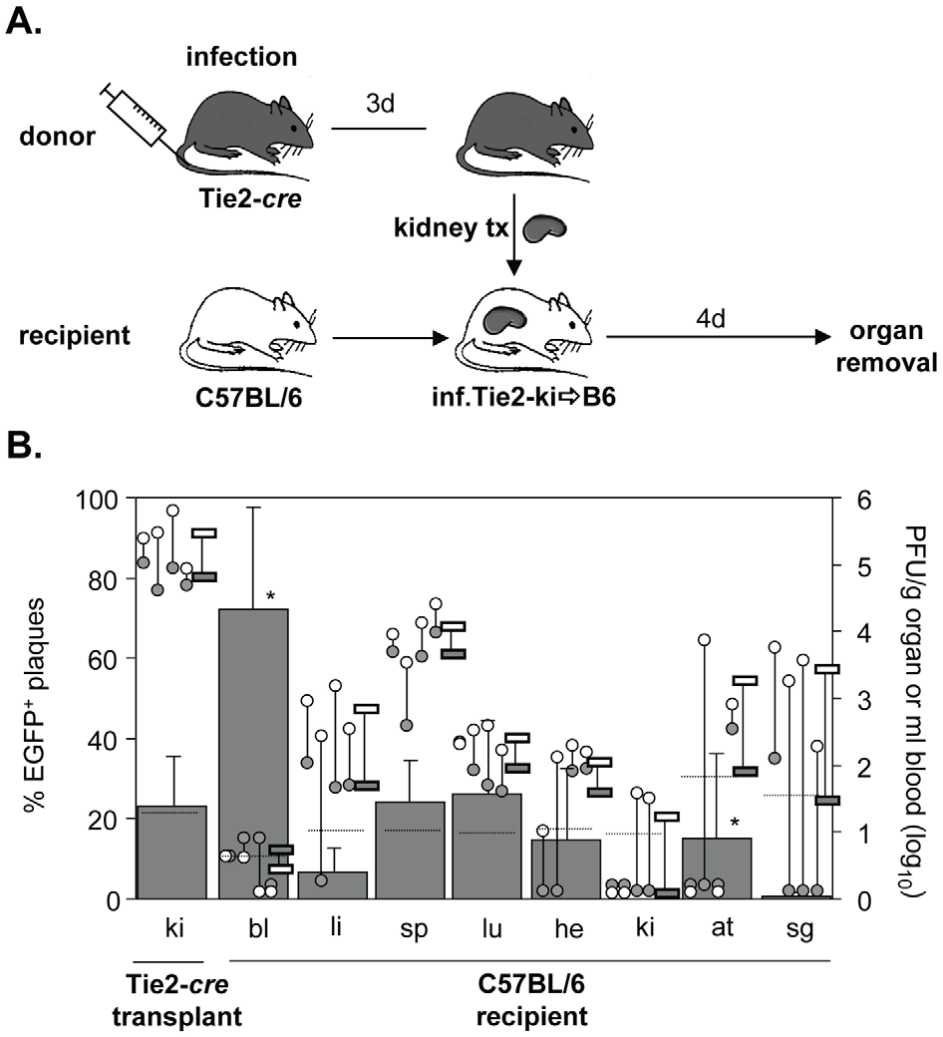
Dissemination of EC- and non-EC-derived MCMV from kidney transplants to non-infected recipients via primary viremia. **A**. Tie2-*cre* mice (n = 4) were infected i.v. with 8×10^5^ PFU MCMV-flox. Three days after infection kidneys were transplanted into non-infected C57BL/6 mice. Four days after transplantation blood was taken and organs were collected from recipients and analyzed. **B**. Data are depicted as described in Fig. 1A. Virus load of MCMV-flox (open circles) and MCMV-rec (grey circles) per gram organ or ml blood for individual mice are shown. Grey columns indicate the mean percentage of EGFP^+^ plaques (MCMV-rec compared to MCMV-rec plus MCMV-flox). Abbreviations: he = heart; bl = blood; li = liver; sp = spleen; lu = lungs; ki = kidney; at = adipose tissue; sg = salivary glands.

Next, we studied dissemination of MCMV following kidney transplantation. Kidneys represent the majority of transplanted organs in medicine. Similar to heart transplantation, the transplantation of kidneys from seropositive donors to seronegative recipients is associated with a high risk to develop CMV-related complications [28], [29], [30]. Four days after heterotopic transplantation of infected kidneys of Tie2-*cre* mice into non-infected C57BL/6 mice, recipient organs were analyzed for the presence of disseminated virus (Fig. 2A). In contrast to the heart, only about 20% of virus within transplanted kidneys was recombined (Fig. 2B). This low contribution of EC-derived virus to total virus load in kidney is in line with previous observations [24]. As recombination rates were similar in Tie2-cre and Tek-cre mice, we believe that the low proportion of MCMV-rec in Tie2-*cre* kidneys does not necessarily indicate a low recombination efficiency in renal EC but may rather result from an alternative mode of virus entry into kidney tissue bypassing the vascular endothelium for replication in other cell types, one candidate being kidney epithelial cells. The relative levels of virus titers in liver, spleen, and lungs were comparable to those observed following heart transplantation. Yet, the percentage of recombined, EC-derived virus in most organs essentially mirrored the situation in the transplanted kidney, and there was no preferential dissemination of EC-derived virus (∼20%). Collectively, the findings after transplantation of two different organs did not support the hypothesis of a predominant role of EC in virus dissemination during the first four days of infection. Only in blood a significantly higher proportion of MCMV-rec was found on day four post kidney transplantation in 3 out of 4 mice. However, as the absolute virus titers were close to the detection limit, any interpretation has to be seen with caution.

### Minor contribution of EC-derived virus from the transplanted heart to virus dissemination in the systemically infected host

**Figure 3:**
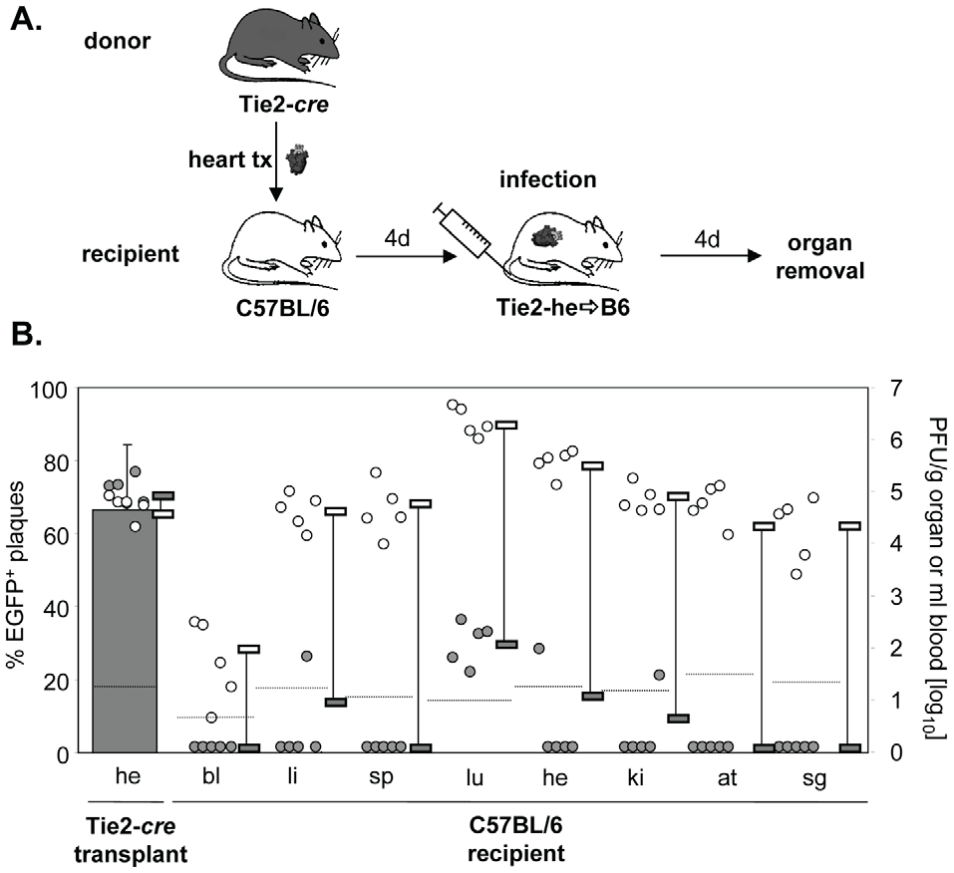
Little dissemination of EC-derived MCMV from heart transplants via secondary viremia. **A**. Hearts from Tie2-*cre* mice were transplanted into C57BL/6 mice (n = 5). Four days later recipients were infected i.v. with 8×10^5^ PFU MCMV-flox. Blood was taken and organs were collected four days after infection and analyzed. **B**. Data are depicted as described in Fig. 1A. Virus load of MCMV-flox (open circles) and MCMV-rec (grey circles) per gram organ or ml blood for individual mice are shown. Grey columns indicate the mean percentage of EGFP^+^ plaques (MCMV-rec compared to MCMV-rec plus MCMV-flox). Abbreviations: he = heart; bl = blood; li = liver; sp = spleen; lu = lungs; ki = kidney; at = adipose tissue; sg = salivary glands.

In the preceding experiments, the systemic infection originated from a pre-infected transplanted organ. Next, we studied the contribution of EC of a transplanted Tie2-*cre* ^+^ heart to virus dissemination during the situation of systemic infection of C57BL/6 recipients. Under these conditions, all organs, including the transplanted heart, become infected simultaneously. Thus, MCMV-rec, wherever found, must have originated from ECs of the transplant. Note that under such conditions the infection of the transplant does not have a head start. Four days after transplantation mice were systemically (i.v.) infected with MCMV-flox and four days later they were sacrificed and virus titers determined (Fig. 3A). As expected, the majority of virus in the transgenic heart transplant was found to be recombined (Fig. 3B). Despite this, we observed only very little dissemination of EGFP+ EC-derived MCMV from the transplant to infected recipient organs. In the lungs, some MCMV-rec was found at very low numbers, four orders of magnitude lower than MCMV-flox, whereas in other organs MCMV-rec was at or below detection limit. This result is in stark contrast to the dissemination from the infected transplant (Fig. 1) where 70-90% of virus progeny in recipient organs were EC-derived. It is important to note that total virus titers in both the endogenous and transplanted heart were very similar (Fig. 3B), indicating efficient vascularization of the heterotopic heart transplant after surgery. This excludes an impaired blood flow as a presumed reason for the observed poor dissemination of recombined virus. We thus conclude that virus dissemination from the heart plays a negligible role during systemic infection.

Another striking difference between virus dissemination from both transplanted heart and kidney as compared to systemic (i.v.) infection was the extent of virus production in different organs. In contrast to i.v. infection, which resulted in peak titers in the lung and high titers in heart, kidney, liver, spleen and adipose tissue (Fig. 3), virus dissemination from transplanted heart and kidney (Figs. 1 and 2) resulted in peak titers in spleen but significantly lower titers in liver and lung, and in almost no virus detectable in the endogenous heart, kidneys, and adipose tissue. This cannot simply be explained by organ specific differences in virus production kinetics [24] but rather indicates a qualitative difference in virus dissemination between systemic (i.v.) infection (free virus) and transplant-mediated infection.

### Limited colonization of the heart by EC-derived virus via secondary viremia

**Figure 4:**
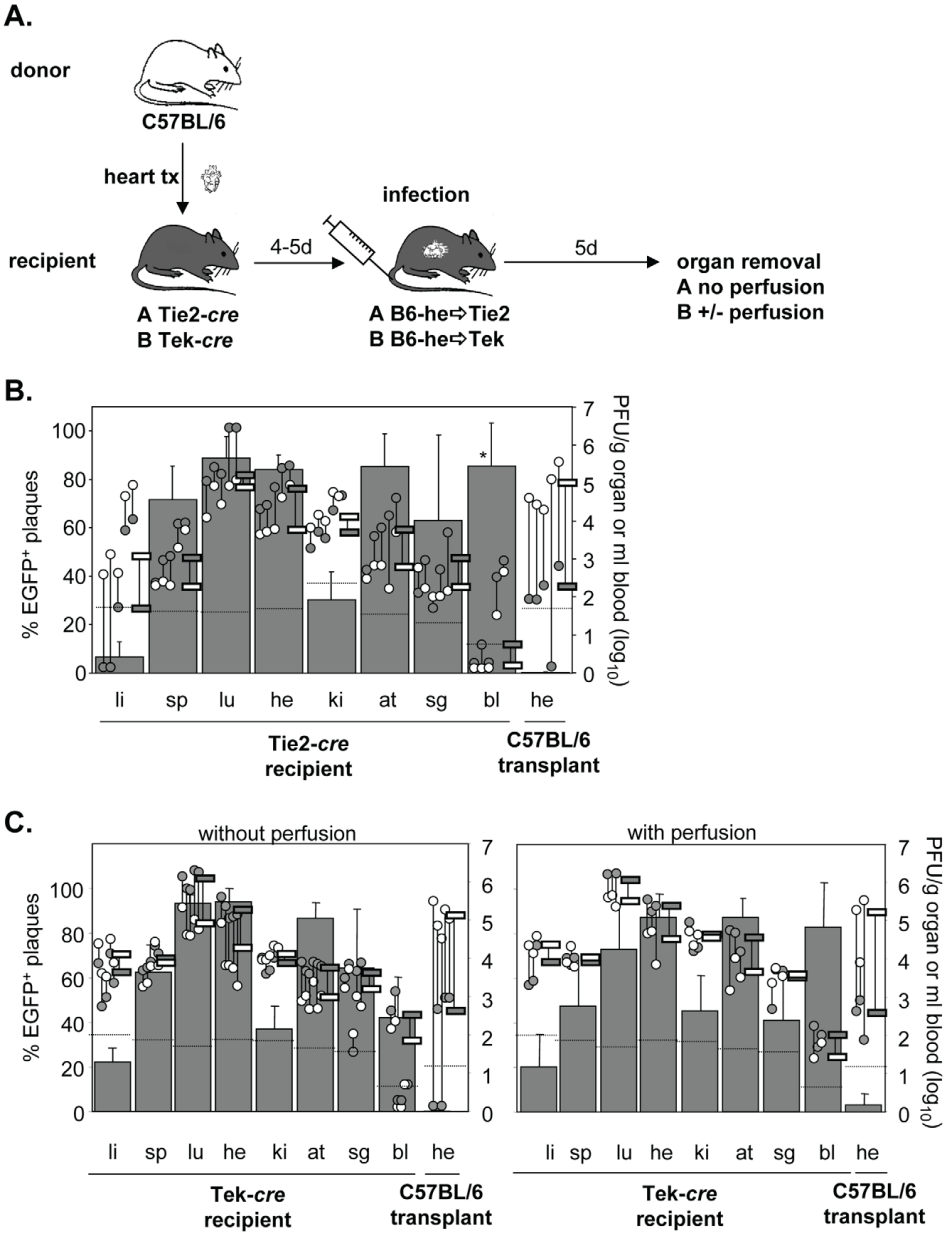
Minor colonization of heart transplants by EC-derived MCMV via secondary viremia following systemic infection. **A**. Hearts from C57BL/6 mice were transplanted into Tie2-cre (n = 5; **B**.) or Tek-cre mice (**C**.) and recipients were infected i.v. with 8×10^5^ PFU MCMV-flox four to five days later. Five days after infection blood was taken and organs were collected from recipients and analyzed. Data are depicted as described in Fig. 1A. In (**C**.) organs were removed from non-perfused (left hand panel, n = 5) or perfused (right hand panel, n = 3) recipient mice. Data of non-perfused and perfused groups were obtained in different experiments. Abbreviations: he = heart; bl = blood; li = liver; sp = spleen; lu = lungs; ki = kidney; at = adipose tissue; sg = salivary glands.

During systemic infection following transplantation of a *cre*-positive heart to a *cre*-negative mouse no significant contribution of virus dissemination from the heart transplant to other organs was observed. To study not only cardiac EC but EC in general as a source of virus dissemination, Tie2-*cre* or Tek-*cre* recipient mice received a non-transgenic heart. Recipients were then infected i.v. with MCMV-flox (Fig. 4A). As expected, the host organs showed the previously described organ-specific contribution of EC to total virus load [24]. Specifically, in liver the bulk of virus is derived from hepatocytes as we recently showed using Alb-*cre* mice selectively expressing Cre recombinase in hepatocytes [24], whereas the cell type producing the bulk of virus in kidney remains to be determined. In all other organs, >60% of virus proved to be EC-derived. Yet, although the transplanted heart contained a total virus load comparable to that of the endogenous heart, there was only a minute (about 1%) contribution of recombined virus to the amount of virus in the transplant (Fig. 4B). We repeated the experiments in Tek-*cre* mice, another mouse line transgenic for *cre* in EC, and obtained essentially the same results (Fig. 4C). To confirm that this small contribution of MCMV-rec to the infection of a heart transplant was truly due to virus seeding to the organ and not just reflected virus present in the circulation, organ perfusion was performed in order to flush out blood cells prior to analysis (Fig. 4C). In any case, the data revealed an only minute dissemination of EC-derived virus via secondary viremia following systemic infection.

### Dissemination of EC-derived MCMV from an infected host to a transplant

**Figure 5:**
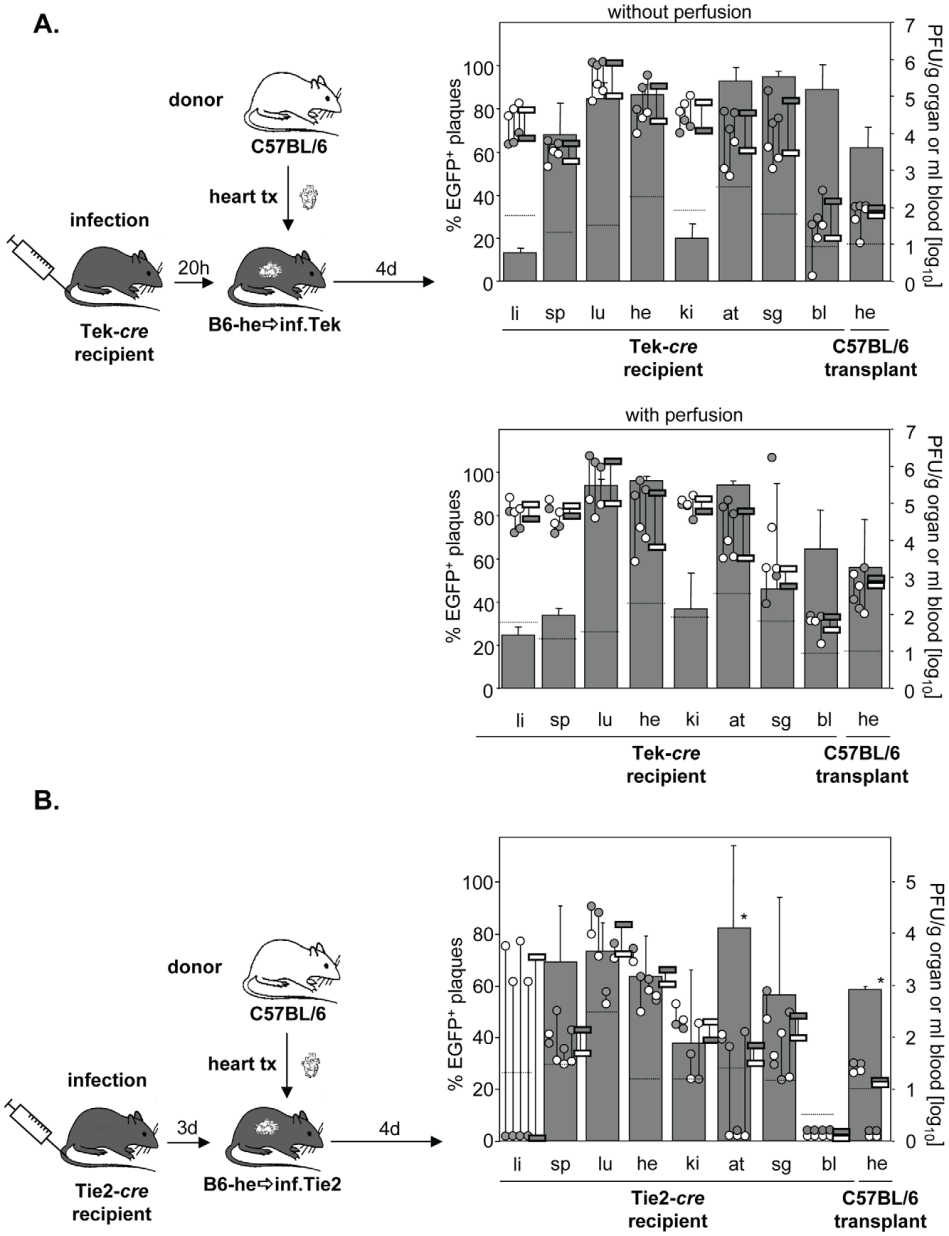
Minor colonization of heart transplants via secondary viremia. Tek-*cre* mice (n = 3; **A**) or Tie2-cre (n = 4; **B**) were infected i.v. with 8×10^5^ PFU MCMV-flox and received a heart transplant derived from non-infected C57BL/6 mice 20 hours (**A**) or three days (**B**) later. Four days after transplantation blood was taken and organs were collected from recipients and analyzed. Data are depicted as described in Fig. 1A. In (**A**), recipient organs were either not perfused (upper panel, n = 3) or were perfused (lower panel, n = 3) before collecting organs. Data of non-perfused and perfused groups were obtained in different experiments. Abbreviations: he = heart; bl = blood; li = liver; sp = spleen; lu = lungs; ki = kidney; at = adipose tissue; sg = salivary glands.

The low degree of dissemination of MCMV-rec into the heart could be the result of two scenarios. We expected that the immune response induced by systemic infection actively prevented secondary import of EC-derived virus into the transplant. Alternatively, after initial virus seeding by systemic (i.v.) infection, local virus production might simply outnumber secondary import of EC-derived virus. To address this issue and to initiate the activation of immune functions, systemic infection was performed prior to transplantation. Specifically, Tie2-*cre* or Tek-*cre* mice were first i.v. infected with MCMV-flox and only then received heart transplants of non-infected C57BL/6 mice either 20 h or 3 days after infection (Fig. 5A/B). Strikingly, systemic infection prior to transplantation increased the relative contribution of EC-derived virus in the transplant from ∼5% (Fig. 4B) to ∼60% independent of the time delay between infection and transplantation (Fig. 5A/B). This average of about 60% MCMV-rec reflects the average contribution of MCMV-rec in the organism in general. However, total virus titers in the heart transplant were 100- to 1000-fold lower than in both the endogenous heart exposed to i.v. infection as well as the hearts transplanted prior to i.v. infection (Fig. 4B/C). It is important to note that the absolute amounts of recombined virus in the heart transplants (grey circles in Fig. 5A/B) were on the same level with those observed following i.v. infection after heart transplantation (grey circles in Fig. 4B/C). Similar results were obtained after perfusion of recipient organs thus demonstrating that the detected virus was not blood-borne but was indeed produced within the transplanted organ.

Total titers in the transplant decreased when transplantation was delayed from 20 h to 3 days after infection, reflecting the situation at day 5 and 7 p.i., respectively. In two animals transplanted three days after infection, virus titers in the heart transplant even fell below the detection limit of 10 PFU/g organ, probably reflecting enhanced control by the host immune system at day 7. This is supported by the relatively low virus titers in spleen, kidneys and adipose tissues as well as by the lack of detectable virus in blood (Fig. 5B).

In conclusion, we were surprised to see that ongoing virus replication and the accompanying immune response in the transplanted heart did obviously not alter the absolute amount of EC-derived virus originating from recipients' tissues by secondary viremia. These data demonstrate that virus dissemination between organs – originating from both endothelial and non-endothelial cells – has only minor effects on organ viral load following systemic infection.

## Discussion

One hallmark of CMV infection is the ability of the virus to infect many cell types and tissues from which again the virus may spread. Apparently, immune control defines to which extent this potential is realized in a given scenario. Therefore, the various clinical conditions need to be considered to explain CMV pathogenesis. Blood specimens play an important role in CMV diagnostics. Proper usage of the information gained by this analysis should monitor or even predict events that happen in organs. However, it is currently unclear under which conditions CMV is spread via blood. Fenner *et al.* were the first to propose a two-step dissemination model for systemic virus infections. Primary viremia transports the virus from the site of entry to liver and spleen where the virus replicates. Secondary viremia then causes dissemination from liver and spleen throughout the body [31]. This model became widely accepted for many viruses to this day, including CMV [6], [32]. Yet, the original model was developed prior to any knowledge on innate immunity control functions and did thus not consider major factors in virus host defense. Recently, we challenged this view for CMV infection in the mouse model with respect of the role of the liver. Virus produced in hepatocytes is locally disseminated to other cell types but is not distributed from the liver to other organs via secondary viremia [24].

In the present study the vascular EC were analyzed for their claimed role in contributing to the CMV load in organs, and in disseminating the virus via primary or secondary viremia. Our salient findings are as follows: EC-derived virus significantly, ∼50% of the body virus pool, contributes to total virus load during acute infection. This contribution was quantified for the first time for the major organs. Yet, there was obviously no preference for dissemination of EC-derived virus over virus produced by other cell types. In addition, and similar to hepatocyte-derived virus, EC-derived virus was poorly disseminated via secondary viremia. These data raise doubts on the indiscriminate applicability of the primary and secondary viremia concept to virus infections in general.

Properties of EC have enticed scientists to consider them as key production sites for virus dissemination, as they may release free virus particles directly into the blood stream or may detach from the vessel wall and transfer virus to other organs via the blood stream [14], [15]. Moreover, EC could transfer the virus by contact to other cell types such as monocytes or granulocytes [33], [34], which would then disseminate the EC-derived virus to other organs [12], [35]. On the other hand, EC-derived virus may also spread to underlying parenchyma and leave the organs via the draining lymph nodes to eventually reach the blood circulation via the thoracic duct. As heterotopic, abdominal transplants are not connected to lymph vessels, exiting virus would enter the peritoneal cavity that is drained by the mediastinal lymph nodes. This lymphatic dissemination route was recently described after intraperitoneal MCMV infection [36] and is also generally accepted as dissemination route after local infections, including intraplantar infection with MCMV [37].

Here, we provide the first quantitative analysis of organ- and cell type-specific virus dissemination. From an infected organ EC-derived virus readily disseminated to the other, uninfected organs. In the specific cases shown here, the infected transplanted organ (heart or kidney) created the condition of a primary viremia initiating from a defined source. EC-derived virus remained a stable fraction in both heart (∼80%) and kidney (∼20%) throughout the first week of infection [24], thereby providing a constant supply of virus. Yet, the percentage of EC-derived virus that disseminated to other organs essentially mirrored the relative contribution of EC in the transplanted organ. Thus there was no preferential seeding of EC-derived virus.

Infected EC might detach from the vessel wall and circulate. In fact, HCMV-infected EC were considered as a parameter for the diagnosis of HCMV organ involvement and for the study of the pathogenesis of disseminated infection [16]. This conclusion was originally based on the finding of two symptomatic patients with a high load of infected circulating EC, but experimental evidence for EC-derived virus colonizing other organs was missing so far. In the mouse model we now provide a nuanced view on the role of EC in virus dissemination. If the infected heart transplant is the source of primary infection, then EC-derived virus is readily disseminated, but without preference. During secondary viremia, however, there is only negligible import of EC-derived virus into the transplant as well as export from the transplant, and this is apparently independent of the extent of ongoing virus replication, associated inflammation, and immune control.

Do our findings formally exclude any prominent role of EC-derived virus? The answer is both yes and no. Yes, we can exclude this role in the mouse model and for the temporal conditions of our experiments. Unfortunately, the more time passes after initial infection of the animal the definition of virus as being EC-derived virus becomes more and more indirect. EC-derived virus progeny keeps the marker independent of the cell type in which the virus replicates in further replication rounds. Thus, with our experimental setup we cannot study later phases of infection when other conditions of virus productivity and dissemination may prevail. However, according to our previous experience, second and third replication rounds contribute less and less to the viral load in the immune competent host due to the onset of immune control [24]. We have not yet studied the situation of the immune deficient host for the EC progeny. For the hepatocyte-derived progeny, however, we know that immune suppressive regimens, even if combined, do not lift the strong dissemination block [24].

Nevertheless, by comparing the virus titers in different organs following transplant-derived and i.v. infection, we observed striking differences. Systemic (i.v.) infection with tissue culture produced virus preparations resulted in a uniform distribution of virus to many organs, whereas transplant-derived virus appeared to preferentially colonize spleen, lung and liver but not heart, adipose tissues and kidneys. This cannot be explained by known differences in organ specific virus kinetics. Therefore, cell-free virus, which is usually used for experimental infection, is apparently able to efficiently colonize all organs, whereas virus leaving an infected organ via a natural route reveals a different kind of spread. What could be the cause of the difference between i.v. infection with a solution enriched in isolated virions and the spread of infection from an infected organ? The most plausible explanation is that the virus leaving an infected organ during systemic infection is predominantly transported in a cell-associated manner. Yet, this difference in organ and tissue distribution shows no preference for EC-derived virus and is altogether marginal with respect to total virus load in an organ.

## Materials and Methods

### Ethics statement

This study was carried out in strict accordance with the recommendations and guidelines for the care and use of laboratory animals according to Tierschutzgesetz (TierSchG, BGBI S. 1105; 25.05.1998). All animal experiments were approved by the responsible state office (Regierung von Oberbayern) under permit number 55.2-1-54-2531-19-07.

### Cells and mice

M2-10B4 (CRL-1972; ATCC) and BALB/c-derived mouse embryo fibroblasts (MEF) were grown as described previously [38]. Transgenic Tie2-*cre* [39] and Tek-*cre* [11] mice were housed at the animal facility of the Max von Pettenkofer-Institute under specified-pathogen-free (SPF) conditions. *Cre*-transgenic mouse strains were maintained on the C57BL/6J background. Experiments were performed with gender matched pairs of mice at 3 to 12 months of age. C57BL/6J mice were obtained from Janvier. Tek-*cre* mice were obtained from Jackson Laboratories (nr. 4128).

### Viruses and infection of mice

All viruses were derived from the molecular clone pSM3-fr [40]. Mutant virus (MCMV-flox) was generated as described [24]. Viruses were propagated on M2-10B4 cells and purified as described [41]. Virus quantification was done by standard plaque titration assay on MEF. Mice were infected intravenously (i.v.; into a tail vein) with 8×10^5^ PFU in a volume of 300 µl.

### Organ transplantations

Syngeneic transplantations of hearts or kidneys were performed between C57BL/6 mice and Tie2-*cre* or Tek-*cre* mice that were maintained on the genetic background of C57BL/6 mice.

Heart transplant model: Abdominal-heterotopic cardiac transplants were performed, as previously described [42]. Briefly, the ascending aorta of the graft was anastomosed to the abdominal aorta of the recipient and the pulmonary artery to the inferior vena cava while the pulmonary veins were ligated. The graft function was assessed by daily palpation.

Kidney transplant model: The murine kidney transplantation was performed as described previously [43]. Briefly, the left kidney of the donor was harvested and transplanted into the recipient. The kidneys of the recipients were not removed. A bladder patch was anastomosed to the recipient's bladder. No signs of rejection due to Cre expression by EC of the transplants or by EC of the recipient were seen throughout the experiments excluding host versus graft or graft versus host reactions, respectively.

### Virus determination in organs

Virus load in organs was determined by plaque assay as described previously [24] with the modification that blood samples were sonicated before they were added to MEF in a volume of 10 µl per well. The numbers of MCMV-rec and MCMV-flox plaque forming units (PFU) were determined from organ homogenates after 4 days and from blood after 5 days using a fluorescence microscope (Olympus). Only plaques visible in bright field were considered for the calculation. PFU were calculated per ml of blood or g of organ.

### Perfusion of recipients and heart transplants

Mice were anaesthetized and the peritoneal cavity was opened. After injection of 50 µl of heparin into the inferior vena cava, abdominal aorta and vena cava were cut cranially of the transplant. After all organs were perfused with 5 ml PBS via the vena cava the heart transplant was removed and perfused separately with 3 ml PBS.

### Statistical analysis

The percentage of MCMV-rec compared to total virus organ load per group, mean values, and standard deviations were determined.
